# Radiobiological impact of dose calculation algorithms on biologically optimized IMRT lung stereotactic body radiation therapy plans

**DOI:** 10.1186/s13014-015-0578-2

**Published:** 2016-01-22

**Authors:** X. Liang, J. Penagaricano, D. Zheng, S. Morrill, X. Zhang, P. Corry, R. J. Griffin, E. Y. Han, M. Hardee, V. Ratanatharathom

**Affiliations:** Department of Radiation Oncology, University of Arkansas for Medical Sciences, 4301 W. Markham St., #771, Little Rock, AR USA; Department of Radiation Oncology, University of Nebraska Medical Center, 42nd and Emile, Omaha, NE USA; Department of Radiation Oncology, University of Texas MD Anderson Cancer Center, Houston, TX USA

**Keywords:** Combined dose-volume and biologically optimized IMRT, AAA and AXB dose calculation algorithms, TCP, NTCP, Fractioned stereotactic radiotherapy, Non-small-cell lung cancer

## Abstract

**Background:**

The aim of this study is to evaluate the radiobiological impact of Acuros XB (AXB) vs. Anisotropic Analytic Algorithm (AAA) dose calculation algorithms in combined dose-volume and biological optimized IMRT plans of SBRT treatments for non-small-cell lung cancer (NSCLC) patients.

**Methods:**

Twenty eight patients with NSCLC previously treated SBRT were re-planned using Varian Eclipse (V11) with combined dose-volume and biological optimization IMRT sliding window technique. The total dose prescribed to the PTV was 60 Gy with 12 Gy per fraction. The plans were initially optimized using AAA algorithm, and then were recomputed using AXB using the same MUs and MLC files to compare with the dose distribution of the original plans and assess the radiobiological as well as dosimetric impact of the two different dose algorithms. The Poisson Linear-Quadatric (PLQ) and Lyman-Kutcher-Burman (LKB) models were used for estimating the tumor control probability (TCP) and normal tissue complication probability (NTCP), respectively. The influence of the model parameter uncertainties on the TCP differences and the NTCP differences between AAA and AXB plans were studied by applying different sets of published model parameters. Patients were grouped into peripheral and centrally-located tumors to evaluate the impact of tumor location.

**Results:**

PTV dose was lower in the re-calculated AXB plans, as compared to AAA plans. The median differences of PTV(D_95%_) were 1.7 Gy (range: 0.3, 6.5 Gy) and 1.0 Gy (range: 0.6, 4.4 Gy) for peripheral tumors and centrally-located tumors, respectively. The median differences of PTV(mean) were 0.4 Gy (range: 0.0, 1.9 Gy) and 0.9 Gy (range: 0.0, 4.3 Gy) for peripheral tumors and centrally-located tumors, respectively. TCP was also found lower in AXB-recalculated plans compared with the AAA plans. The median (range) of the TCP differences for 30 month local control were 1.6 % (0.3 %, 5.8 %) for peripheral tumors and 1.3 % (0.5 %, 3.4 %) for centrally located tumors. The lower TCP is associated with the lower PTV coverage in AXB-recalculated plans. No obvious trend was observed between the calculation-resulted TCP differences and tumor size or location. AAA and AXB yield very similar NTCP on lung pneumonitis according to the LKB model estimation in the present study.

**Conclusion:**

AAA apparently overestimates the PTV dose; the magnitude of resulting difference in calculated TCP was up to 5.8 % in our study. AAA and AXB yield very similar NTCP on lung pneumonitis based on the LKB model parameter sets we used in the present study.

## Introduction

The goal of radiation therapy is to optimize therapeutic ratios by delivering tumoricidal doses to targets while maximally sparing organs-at-risk (OARs). Mostly, the quality of a radiation treatment plan is judged by isodose distribution and dose-volume-histograms (DVH). Typically the biological outcomes in terms of tumor control and normal tissue complication are not estimated when evaluating a plan. Significant progress and contributions to our understanding and modeling of volume effects for both normal and tumor tissues started in the 1980s with the advent of modern three dimensional treatment planning techniques. Models for estimating the tumor control probability(TCP) and normal-tissue complication probabilities (NTCP) were proposed in the late 1980s [1–8]. Even though dose-volume techniques are a mainstay of current clinical treatment planning optimization, biological optimization using complication probability models in intensity modulated radiotherapy (IMRT) planning has shown potential for reducing radiation-induced toxicity [9–11]. The current study used combined biological optimization and dose-volume optimization to take advantage of using radiobiological models and at the same time also keep the “important” dose-volume characteristics. The report of AAPM Task Group 166 [12] recommends that dose-volume constraints and the biologic optimization function be used together for optimization.

In 2005, Eclipse TPS released the Analytical Anisotropic Algorithm (AAA) [13]. AAA is a convolution–superposition-based photon beam dose computation algorithm. This algorithm was quickly and widely adopted for clinical use. More recently, Varian Eclipse TPS implemented another dose calculation algorithm, Acuros XB Advanced Dose Calculation (AXB), which uses a deterministic grid-based Boltzmann equation solver (GBBS or the discrete ordinates method). The GBBS [14, 15] explicitly solves the linear Boltzmann transport equation (LBTE), which is the governing equation that describes the macroscopic behavior of ionizing particles (neutrons, photons, electrons, etc) as they travel through and interact with matter. The GBBS then iteratively solves the radiation transport problem within specified volumes to compute radiation doses. AXB was first published by Vassiliev et al. [16] and has been considered to be similar to classic Monte Carlo methods for accurate modeling of dose deposition in heterogeneous media [16–18].

Among the numerous studies comparing the dosimetric differences between plans calculated with conventional algorithms (pencil beam type and convolution-superposition type) vs. with advanced algorithms (Monte Carlo type and GBBS type) [19–26], lung SBRT has been shown as the treatment where the differences due to dose algorithms are among the most significant, hence necessitating the adoption of the more advanced algorithms. This is due to the low density lung tissue and the high risk of normal tissue toxicity in hypofractionated treatments like SBRT. Compared with the very large dosimetric differences found between pencil beam type algorithms and advanced algorithms, smaller differences were seen between convolution-superposition typealgorithms such as AAA and advanced algorithms. Improved accuracy with advanced algorithms was always observed and deemed necessary in some cases. Pertaining to the two dose algorithms investigated, studies [19, 20] have illustrated that AXB is more accurate in modelling the radiation transportation and dose deposition in the patient. However, those studies were focused purely on dosimetric comparisons between AAA and AXB algorithms. The impact of these two algorithms on biological indices has not been thoroughly studied. To date, the radiobiological impact of AAA and AXB dose computation algorithms on lung tumor treatment plans, where the impact of dose algorithms would be prominent due to the low density lung tissue, has not been published. Furthermore, planning techniques in the existing literature investigating dosimetric differences between the conventional and advanced dose algorithms on lung SBRT were predominantly based on physical dose volume constraints. In this paper, we have retrospectively planned 28 stereotactic body radiation therapy (SBRT) non-small-cell lung cancer (NSCLC) patients using combined dose-volume optimization and biological optimization provided by a Varian Eclipse (Varian Medical Systems, Palo Alto, CA) planning system (V11). Dose computation was performed alternatingly with AAA (V11) and AXB (V11) algorithms on these plans optimized with AAA. The tumor control probability (TCP) and normal tissue complication probability (NTCP) on normal lung tissue (pneumonitis ≥ 2) from AAA and AXB plans were evaluated using the Eclipse biological evaluation module (V1.4).

## Materials and methods

This study was approved by the University of Arkansas Medical Science Institutional Review Board (FWA00001119).

### Treatment planning

Computed tomography (CT) data from 28 patients with Stages I or II inoperable NSCLC previously treated using SBRT were selected for this study. We have divided the patients into two categories based on tumor location; peripheral and centrally located. This distinction was made due to the fact that the initial experience of SBRT in inoperable lung cancer reported increased toxicity for centrally located tumors [27]. A separate cooperative trial was then designed to explore different fractionation schemes for centrally located tumors [28]. Centrally located tumors are those located either within 2 cm of the airway (6 patients), or touch the pericardial pleura (2 patients), or adjacent to mediastinum (1 patient). The rest of the tumors were considered peripheral (19 patients). Patient characteristics are summarized in Table 1. A four-dimensional computed tomography (4DCT) was acquired for each patient. Maximum intensity projection (MIP) and average intensity projection CTs were reconstructed from the 4DCT. Internal target volume (ITV) was contoured using the MIP by one physician, and planning target volume (PTV) was generated by applying a 0.5 cm isotropic margin to the ITV. Each of the following structures was contoured using the average intensity projection CT by the same physician for every patient: bilateral lungs excluding ITV, spinal cord, esophagus, and heart. A chest wall (CW) structure was also contoured as a 2 cm two-dimensional expansion of the ipsilateral lung excluding the lung volume and the mediastinal soft tissue as described by Mutter et al [29]. The treatment planning was carried out on the average intensity projection CT image set. In order to obtain a conformal dose distribution, two ring structures around the PTVwere also generated. These rings are pseudo planning structures used in dose-volume optimization to conform dose to the target and reduce dose to normal tissue. Ring1 was defined as a 1 cm width ring structure with a 4 mm gap to the PTV. Ring 2 was defined from outward of Ring1 to the body surface and extended 3 cm superior/inferior to the PTV.

**Table 1:** Patient characteristics

Characteristics	
Patients	28
Sex, Male/Female	20/8
Median age (range, yrs)	73 (60,88)
Tumor position, peripheral/central	19/9
Median PTV size (range, cc)	
Peripheral	45.6 (15.3, 107.3)
Central	62.3 (19.0,144.9)

**Fig. 1:**
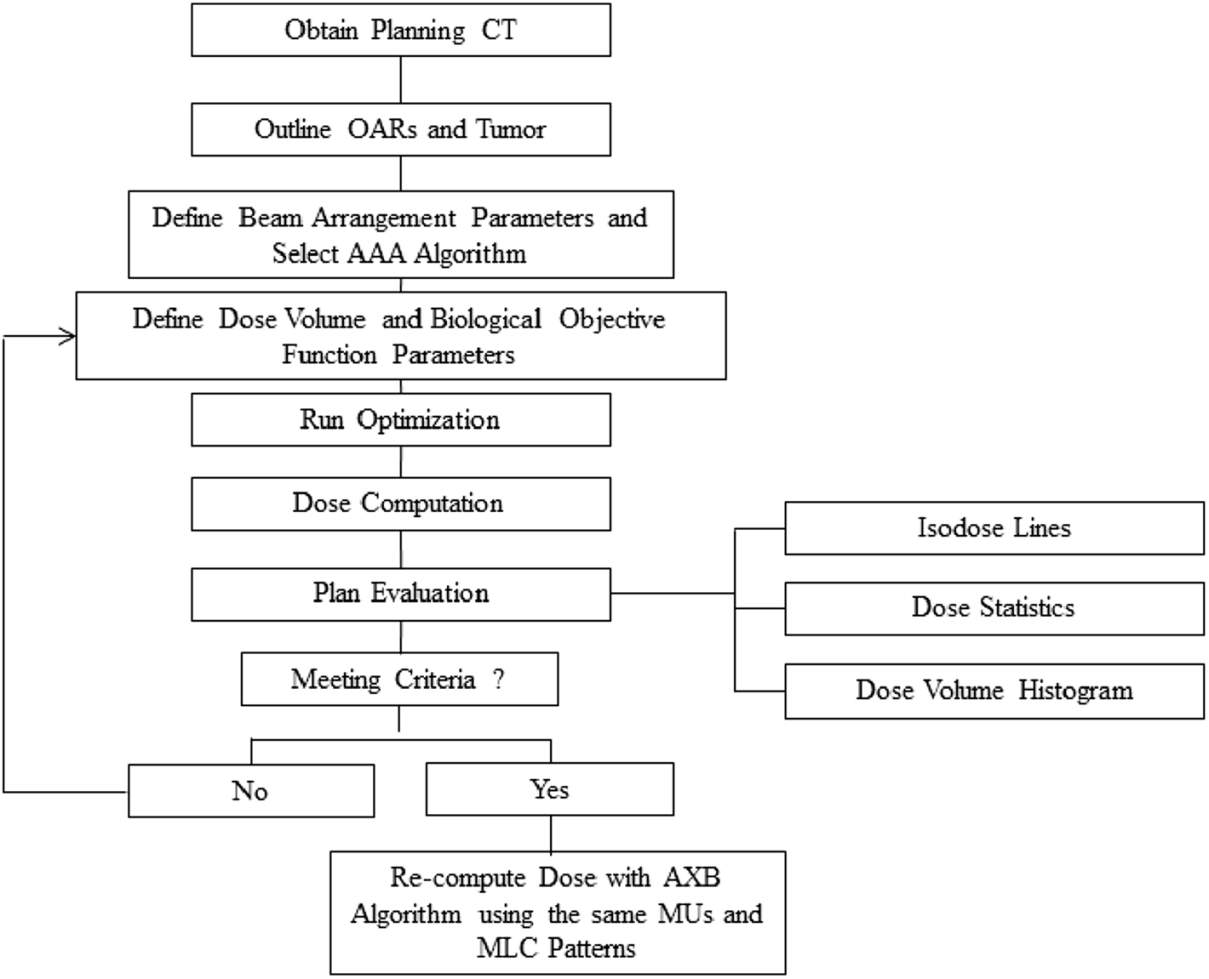
Process flow diagram from CT acquisition to final AAA and AXB plans

These 28 patients previously treated with SBRT were retrospectively planned using Varian Eclipse TPS (V11) with an IMRT sliding window technique. The total dose prescribed to the PTV was 60 Gy with 12 Gy per fraction. Plans were generated on each patient using 9 coplanar 6 MV beams using a True Beam with an HD120 MLC (Varian Medical Systems, Palo Alto, CA). The beam angles were chosen to best cover the PTV, while maximally sparing lung and other critical structures. The isocenter was placed at the center of the PTV. All plans used combined dose-volume histogram and radiobiological optimization to generate the optimal fluence map. The starting dose-volume and biological optimization cost function parameters used in the present study are summarized in Tables 2 and 3. After the initial optimization run, each plan was evaluated and fine-tuned by adjusting the physical dose constraints according to the desired dose distribution. After obtaining the optimized fluence map, the MLC leaf motion and final dose to water were computed using AAA. The treatment plan was normalized such that 95 % of the PTV is covered by the prescribed dose. The plan was evaluated by the same physician in order to ascertain that it met the institutional OAR dose constraints as listed in Table 4. Finally the plan was recalculated using AXB with dose to medium using the same MU and MLC patterns. The calculation grid was set at 0.25 cm for both AAA and AXB algorithms. A process flow diagram from the CT acquisition to the final AAA- and AXB-calculated plans is shown in Fig. 1.

**Table 2:** Dose-volume cost function parameters used in this study

Dose-volume cost function parameters		
Structure	Function type	Physical Dose (Gy)
PTV	Max Dose	<63
	Min Dose	>60
Heart	Max Dose	<20
Spinal cord	Max Dose	<22
BilatLung-ITV	D12%	<20
Chest wall	D13%	<26
Esophagus	Max Dose	<30
	D30%	<24
Ring1	Max Dose	<52.8
Ring2	Max Dose	<30

**Table 3:** Biological cost function parameters used in this study

Biological NTCP-LKB model parameters						
Structure	Endpoint	D_50_ (Gy)	α/β (Gy)	n	m	References
BilatLung-ITV	Pneumonit-is Grade ≥ 2	30.8	1.3	0.99	0.37	[36, 38]
Esophagus	Esophagitis Grade ≥ 2	51.0	10	0.44	0.32	[50]
Heart	Pericarditis	60.6	2.5	0.64	0.13	[51]
Biological NTCP-PLQ model parameters						
Structure	Endpoint	D_50_ (Gy)	α/β (Gy)	ɣ	s	Reference
Spinal cord	Myelitis necrosis	68.6	3	1.9	4.0	[52]
Esophagus	Clinical Stricture	68.6	3	2.8	3.4	[52]

**Table 4:** Normal tissue dose criteria for evaluation of SBRT lung plans

Structure	Max point dose (Gy)	Max critical volume above threshold	Threshold dose (Gy)
Cord	30	N/A	N/A
Heart	38	<15 cc	32
Esophagus	35	<5 cc	19.5
Bilateral Lung - ITV	N/A	1000 cc	13.5
		1500 cc	12.5

### TCP calculation

The Poisson Linear-Quadatric (PLQ) model was used for estimating the tumor control probability. The PLQ model [6] is derived from the linear-quadratic cell survival model using the Poisson distribution:

Where *ɣ* is the normalized dose–response gradient, *D*_*50*_ represents the dose yielding 50 % TCP for a given end point, and *EQD*_*2*_ is the equivalent dose given in 2 Gy fractions and was calculated using equation 2 [30]:

2 $$EQ{D}_2=D\left(1+\frac{d}{\raisebox{1ex}{$\alpha $}\!\left/ \!\raisebox{-1ex}{$\beta $}\right.}\right)/\left(1+\frac{2}{\raisebox{1ex}{$\alpha $}\!\left/ \!\raisebox{-1ex}{$\beta $}\right.}\right)$$

Where *D* is the cumulative dose and *d* is the dose of a single fraction.

The TCP model parameter values were originally obtained by fitting models on clinical data and are therefore dependent on various factors such as patient group, type of treatment, and dose algorithm etc [31]. The range of published *D*_*50*_ on lung treatment was fairly large; Willner et al. [32] converted the total physical dose to 2-Gy fractionation dose equivalent and reported *D*_*50*_ Values of 74.5 Gy for 24 month local control and Martel et al. [33] studied plans with 1.8 – 2.0 Gy per fraction and reported *D*_*50*_ values of 72 and 84.5 Gy for 24 and 30 month local control on NSCLC. While Guckenberger et al. [34] reported a biologic effective dose *D*_*50*_ of 42.3 Gy for 36 month local control. In the work of Guckenberger et al., the patient population was primarily pulmonary metastases. This may partially explain why in their study, *D*_*50*_ was smaller than the *D*_*50*_*’*s reported by the other groups [32, 33] where the studies were on NSCLC patients. Therefore, the higher end of *D*_*50*_ range may be more applicable to our NSCLC cohort. Table 5 summarizes the TCP parameters that we used in the present study. Here we made an approximation that the physical dose of Martel et al. study is the same as *EQD*_*2*_ since 1.8 – 2 Gy per fraction was used in their study. After all the treatment plans were computed with both AAA and AXB, the plans were evaluated using the Eclipse biological evaluation module (V1.4), where the DVHs were corrected to 2-Gy fractionations according to the LQ model. An α/β of 10Gy was used.

**Table 5:** TCP model parameters that used in the present study

References	Mean dose EQD_2_ (Gy)	Local control (months)	*D*_*50*_ (Gy)	ɣ
Willner et al. [32]	60	24	74.5	3.5
Martel et al. [33]	71	24	72.0	2.0
		30	84.5	1.5

### NTCP calculation

The Lyman-Kutcher-Burman (LKB) model was used for estimating normal tissue complication probability on lung pneumonitis. The LKB model is based on a Probit function [1, 3–5]:

3 $$NTCP(LKB) = \frac{1}{\sqrt{2\pi }}{\displaystyle {\int}_{-\infty}^t} exp\frac{-{t}^2}{2}dt$$

where

4 $$t=\frac{\mathrm{EUD} - {D}_{50}}{m\ .\kern0.5em {D}_{50}}$$

The parameter *m* and *D*_*50*_ represent the slope of the sigmoid dose response curve and the dose for a complication rate of 50 %, respectively. *EUD* is the equivalent uniform dose and is calculated as [35]:

5 $$EUD={\displaystyle {\sum}_i}{\left(\ {v}_iEQ{D_{2,i}}^{\raisebox{1ex}{$1$}\!\left/ \!\raisebox{-1ex}{$ n$}\right.}\right)}^n$$

Where *v*_*i*_ is the partial volume with absorbed dose *EQD*_*2,i*_ and *n* is the dose-weighting factor, which defines the risks associated with partial organ volume uniform irradiation.

In the present study, the NTCP values for lung pneumonitis grade ≥ 2 were calculated using the LKB model. Several studies have reported estimates of the model parameters obtained from different clinical studies. A study from Burman et al. [4] was based on treatment plans in which no density correction was performed. Later, Seppenwoolde et al. [36] and Kwa et. al [37] presented difference model parameters obtained from density corrected treatment plans. We applied these three sets of model parameters in this study to investigate the influence of the model parameter uncertainty on NTCP. In addition, we also studied the influence of α/β ratios by applying two different α/β ratios for normal lung tissue; 1.3 Gy from the recent study of Scheenstra et al. [38] and 3 Gy as the standard normal tissue value.

### Results and discussion

**Fig. 2:**
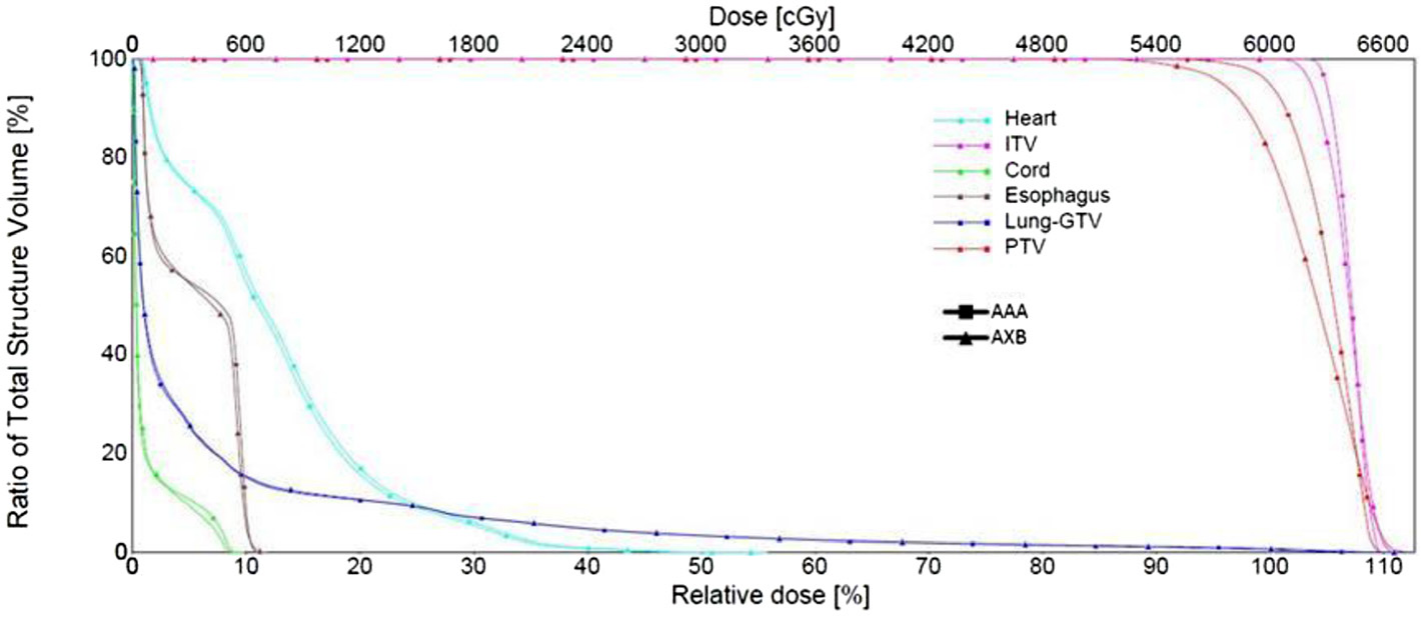
Total physics dose volume histogram of the PTV, ITV and OARs for a typical patient. Dose calculations were performed on an average intensity CT, as generated form a 4D-CT

A comparison of the total physical dose DVHs of the ITV, PTV and OARs for a typical patient plans calculated using the AAA and AXB dose algorithms is shown in Fig. 2. Doses to PTV are generally higher in AAA-calculated plans than AXB-recalculated plans, similar to previous studies [24–26]. A comparison of total physical dose to ITV and PTV calculated using AAA or AXB dose algorithm for both peripheral tumor and centrally-located tumor patients is given in Table 6. A non-parametric Kruskal-Wallis test [39] was used to calculate the p-value with *p <* 0.05 taken as significant. It appears that lower doses to PTV (D_95%_) and PTV (mean) in the re-calculated AXB plans, as compared to AAA plans. The median differences of PTV (D_95%_) were 1.7 Gy (range: 0.3, 6.5 Gy; *p <* <0.01) and 1.0 Gy (range: 0.6, 4.4 Gy; *p <* < 0.01) for peripheral tumor and centrally-located tumor patients, respectively. The median differences of PTV (mean) were 0.4 Gy (range: 0.0 to 1.9 Gy; *P <*0.05) and 0.9 Gy (range: 0.0, 4.3 Gy; *P <*0.05) for patients with peripheral tumors and centrally-located tumors respectively. As shown in Table 6, the difference in the calculated mean dose to ITV is not statistically significant. Here we need to note that our dose distribution was calculated on an average CT generated on a 4DCT scan. There are potential limitations on dose calculation on a static CT of a moving target. On an average CT, a significant fraction of the planning target was represented by low density lung tissue to where the optimizer tried to deliver a higher fluence in order to achieve target dose coverage. Studies on lung SBRT [40, 41] have shown that calculations on static CT underestimated the target dose, as compared to 4D calculations where the dose was computed in a respiratory-correlated CT.To keep the study consistent, the TCP parameters used for analysis in the present study were also obtained from non- respiratory-correlated CT plans.

**Table 6:** Comparison of total physical doses totarget volume calculated using AAA and AXB for peripheral and centrally-located tumor patients

Tumor location	Target (dose metric)	Median dose (range) in Gy		P-values
		AAA	AXB	
peripheral	ITV (mean)	63.7 (62.3, 67.9)	63.9 (60.5, 68.1)	0.86
	PTV (D95%)	60.0 (normalization)	58.3 (53.5,60.3)	4.66E-07
	PTV (mean)	62.9 (61.7, 65.0)	62.2 (60.5, 64.5)	0.04
	PTV (max)	66.0 (63.7, 73.2)	67.0 (64.1, 75.5)	0.19
	PTV (min)	55.2 (51.2, 57.1)	51.3 (37.6, 56.1)	1.19E-05
Central	ITV (mean)	64.7 (63.6, 73.1)	64.1 (61.1, 69.3)	0.31
	PTV (D95%)	60.0 (normalization)	59.0 (55.6, 59.4)	1.00E-4
	PTV (mean)	63.5 (62.8, 67.2)	62.6 (61.0, 66.0)	0.04
	PTV (max)	66.9 (64.8, 86.9)	67.5 (63.9, 84.4)	0.83
	PTV (min)	53.1 (45.1, 57.0)	50.4 (42.3, 53.6)	0.06

**Fig. 3:**
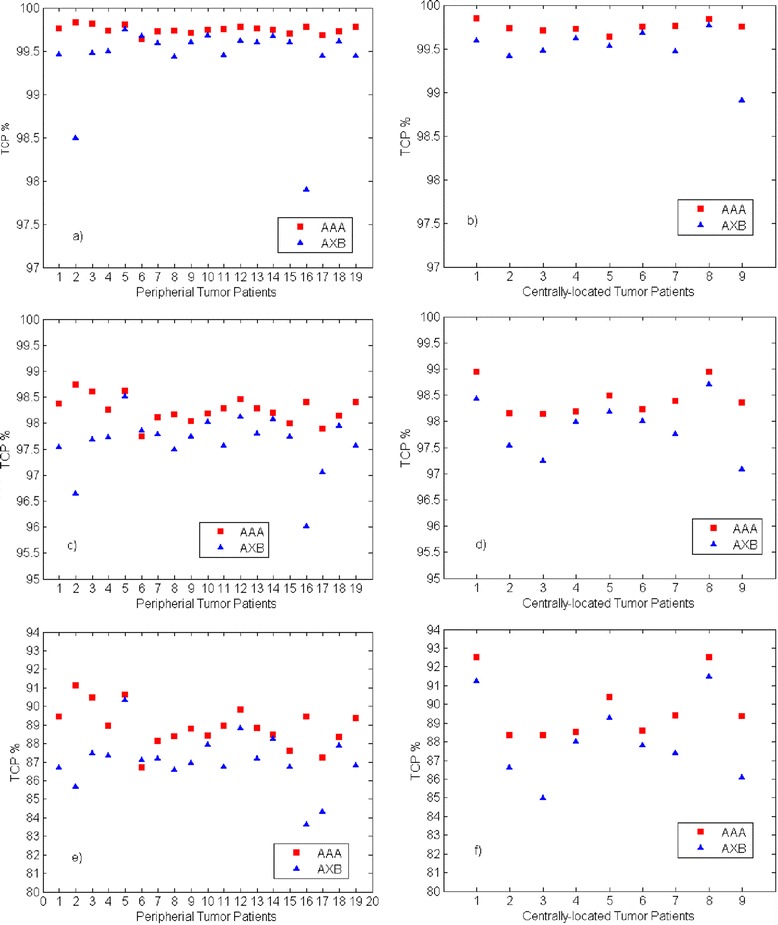
TCP calculated using Martal et al parameter set on PTV of AAA and AXB plans. **a**) and **b**) were calculated using the Willner et al. 24 months local control parameters, **c**) and **d**) were calculated using the Martel et al. 24 months local control parameter set, and **e**) and **f**) were calculated using the Martel et al. 30 months local control parameter set

**Fig. 4:**
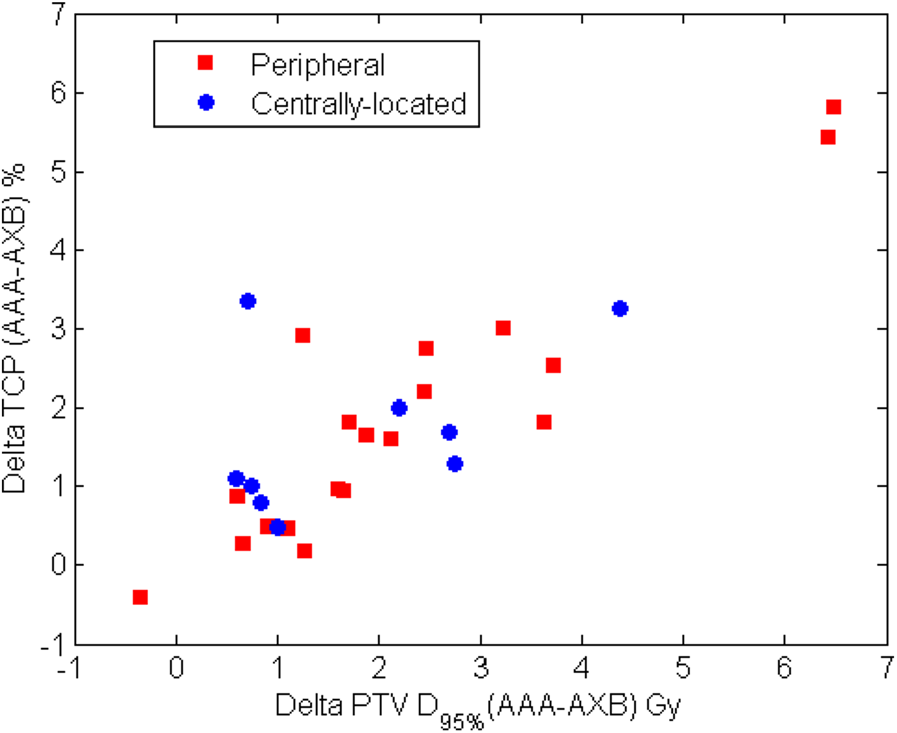
ΔTCP (AAA-AXB) vs. PTV ΔD_95%_(AAA-AXB). Dose displayed here are physical dose

To study the influence of different model parameters on the calculated TCP difference between AAA and AXB plans (ΔTCP), we have applied the Willner et al. and Martel et al. parameter sets for 24 months local control listed in Table 5. Very similar median ΔTCP for 24 months local control were found; for peripheral tumors, 0.5 % (range: 0.1, 2.4 %) and 0.2 % (range: 0.0, 1.9 %), and for centrally-located tumors, 0.5 % (range: 0.2, 1.3 %) and 0.2 % (range: 0.1, 0.8 %) when using Martel et al. and Willner et al. parameter sets, respectively. Figure 3 shows the TCP values from both AAA and AXB plans. The TCP values were found to be lower in AXB-recalculated plans compared with those of AAA-calculated plans. For 30 months local control, the median ΔTCP were1.6 % (range: 0.3, 5.8 %) on peripheral tumor patients and 1.3 % (range: 0.5, 3.4 %) on centrally-located tumor patients. The lower TCP values from the AXB-recalculated plans are associated with a lower PTV dose coverage which is mainly due to the difficulty of the AAA algorithm to properly manage the lateral scattering in low-density media. Figure 4 shows the TCP (30 month local control) differences between AAA and AXB plans (ΔTCP) vs. the total physical dose differences of PTV (D_95%_) in the AAA and AXB plans (ΔD_95%_). It clearly shows that ΔTCP increases as ΔD_95%_ increases. The TCP difference can be as large as 5.8 % on the case with a 6.5 Gy total physical dose (*EQD*_*2*_ of 11.9 Gy) difference in D_95%_. Therefore, we recommend using the most accurate dose calculation algorithm. A smaller ΔTCP for 24 months local control was found compared with ΔTCP for 30 month local control. This may be because the median TCP values for 24 months local control on both AAA and AXB plans were approaching 100 %, even in the AXB-recalculated plans where the PTV dose coverage was lower than the AAA-calculated plans. For peripheral tumors, 97.7 % (range: 96.0, 98.5 %) and 99.6 % (range: 97.9, 99.8 %), and for centrally located tumors, 98.0 % (range: 97.1, 98.7 %) and 99.5 % (range: 98.9, 99.8 %) when using the Martel et al. and Willner et al. parameter set, respectively. Therefore, no substantial ΔTCP can be observed due to the slow slope of the TCP curve at this flat region. While for 30 months local control, the TCP values from AXB-recalculated plans were 87.1 % (range: 83.6, 90.4 %) and 87.8 % (range: 85.0, 91.5 %) for peripheral and centrally-located tumors, respectively. With this level of TCP values, the TCP model was able to show better discriminate between the dose calculations algorithms.

**Fig. 5:**
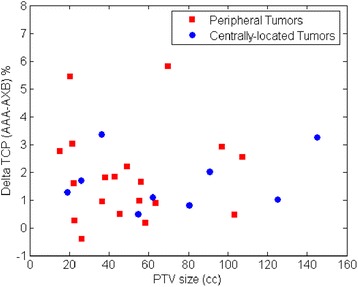
ΔTCP vs. PTV size using the Martel et al. 30 months local control parameter set for both peripheral tumors and centrally-located tumors

**Fig. 6:**
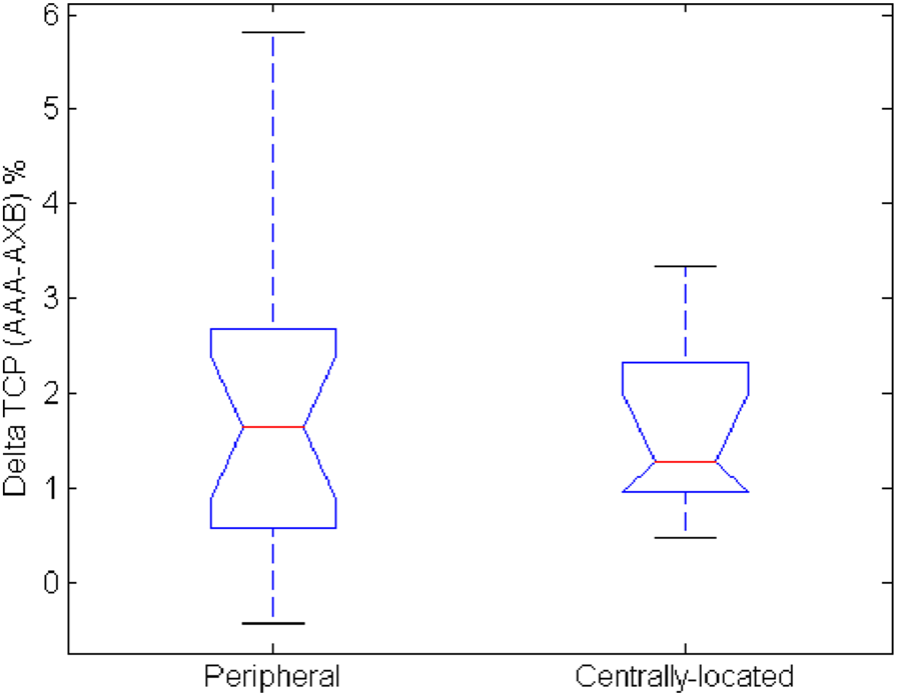
Box plot of ΔTCP comparison between peripheral tumors and centrally-located tumors using the Martel et al. 30 months local control parameter set. The red line represents the median NTCP and the black bars represent the range of the data

In addition, we also studied the relationship between ΔTCP and the PTV size as well as the influence of tumor location on ΔTCP. Figure 5 shows the ΔTCP (30 months local control) vs. PTV size for both peripheral tumors and centrally-located tumors, with no trend observed. Figure 6 shows the box plot of the ΔTCP (30 months local control) comparison between peripheral tumors and centrally-located tumors. The group with peripheral tumors shows a slightly larger median ΔTCP (1.6 %), compared to the group with centrally-located tumors (1.3 %). However, this is not statistically significant with a p-value of 0.86. The dose distribution on PTV depends on a complex set of factors such as the beam angle, the location of tumors relative to low density media in the beam path etc. We have not observed obvious trends of ΔTCP with tumor size and locations within the present study group.

The median and range of Lung pneumonitis NTCP of AAA and AXB plans are listed in Table 7. The model parameters used are also listed in Table 7. Unless otherwise indicated an α/β of 1.3Gy [38] was used. AAA and AXB plans yield very similar NTCP values for both peripheral and centrally-located tumors. The median NTCP and ΔNTCP values are slightly larger in the group with centrally-located tumors, as compared to the group with peripheral tumors. In centrally-located tumors, the critical structures such as esophagus and heart are adjacent to the PTV. In order to spare heart and esophagus, the plan pushes more doses to the normal lung tissue in centrally-located tumor cases. This translates into a slightly sensitive change in NTCPs between the AAA and AXB plans for the group with centrally-located tumors. However, the NTCP difference between the centrally-located tumor group and the peripheral tumor group is not statistically significant (p-value of 0.40).

**Table 7:** Median and range of NTCP on lungpneumonitis grade ≥ 2 for peripheral and centrally-located tumor patients with three different sets of LKB model parameters and two different α/β ratios

	Median(range)%						
	AAA	AXB	(AAA-AXB)	D_50_ (Gy)	n	m	
Peripheral	0.7 (0.2, 5.3)	0.7 (0.1, 5.2)	0.01(-0.04,0.38)	30.5	1	0.3	Kwa [33]
Central	1.8 (0.2, 18.7)	1.7 (0.2, 18.5)	0.03(0.01,0.85)				
Peripheral	2.5 (0.8, 6.0)	2.4 (0.8, 6.1)	0.04(-0.05,0.66)	30.8	0.99	0.37	Seppenwoolde [36] α/β = 1.3Gy [38]
Central	4.6 (0.9, 23.8)	4.4 (0.9, 23.6)	0.07(0.01,0.93)				
Peripheral	2.1 (0.8, 5.8)	2.1 (0.7, 5.7)	0.03(-0.00,0.35)	30.8	0.99	0.37	Seppenwoolde [36] α/β = 3Gy
Central	3.1 (0.8, 13.9)	3.0 (0.8, 13.8)	0.04(0.01,0.47)				
Peripheral	0.2 (0.0, 7.0)	0.2 (0.0, 15.9)	0.01(-0.12,1.10)	24.5	0.87	0.18	Burman [6]
Central	1.5 (0.0, 72.0)	1.2 (0.0, 71.2)	0.19(0.00,5.36)				

**Fig. 7:**
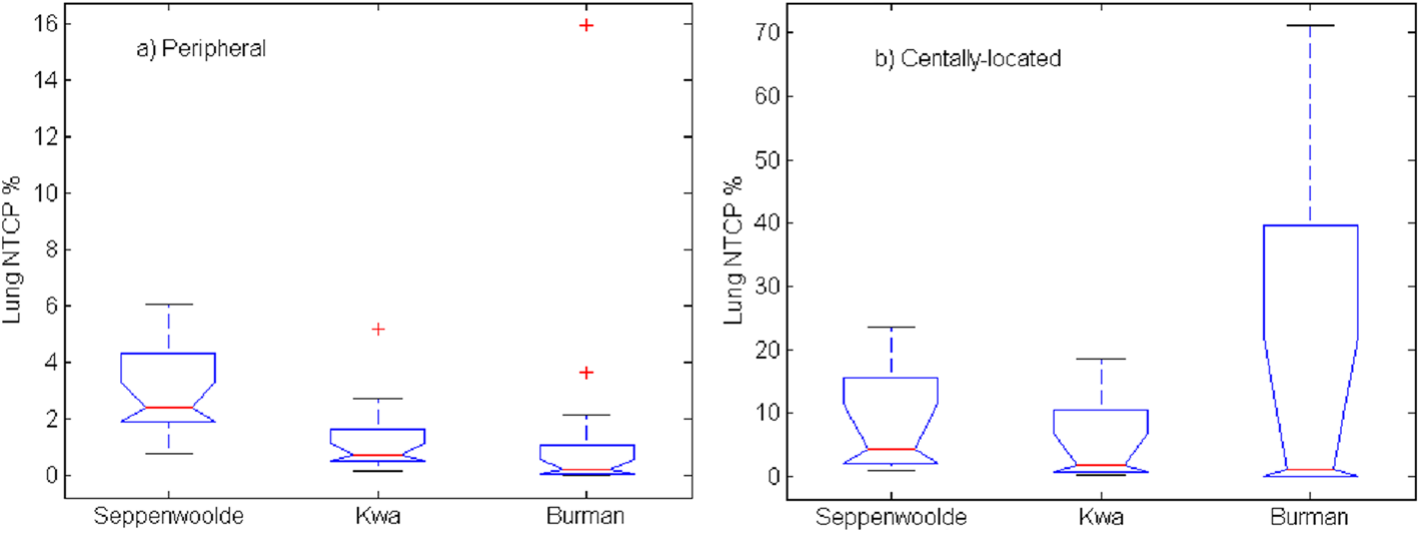
NTCP for lung pneumonitis grade ≥ 2 calculated using the LKB model with three parameter sets. **a** peripheral tumors and **b** centrally located tumors. The red line represents the median NTCP and the black bars represent the range of the data

Figure 7 shows a box plot of the lung NTCP using three different LKB model parameter sets on AXB-recalculated treatment plans. The Burman et al. parameter set predicted the smallest NTCP, the Seppenwoolde et al. parameter set predicted largest NTCP, while Kwa et al. parameter set predicted between the other two parameter sets. Interestingly, in two centrally-located tumor cases, the Burman et al. parameter set predicted much larger NTCP values compares to the other two parameter sets. That is because the Burman et al. parameter set used a much sharper slope of the response curve compared with the other two parameter sets, which results in a more dose-sensitive NTCP prediction. However, when we studied the influence of different model parameter sets on the ΔNTCP, it did not change our conclusion that AAA and AXB plans yield very similar NTCP values. All the ΔNTCP remains <1 % except one outlier, a centrally-located tumor patient with the largest PTV size (144.9 cc), whose ΔNTCP was 5.4 % when using the Burman et al. parameter set to calculate,while values <1 % were obtained when using the other two parameter sets. The small ΔNTCP might be because that the NTCP models cannot discriminate between the dose calculation algorithms at these low NTCP value regions. We also studied the influence of α/β ratios using the Seppenwoolde parameter set with the results also shown in Table 7. Although the smaller α/β ratio (1.3 Gy) predicted slightly larger lung NTCP compared to with the α/β of 3 Gy, again it showed a very minimal influence on ΔNTCP.

The mean lung dose (MLD) has been widely used as a simple and effective metric for probability of pneumonitis [42]. In the present study, we have studied the relationship between the ΔNTCP and the MLD difference between AAA and AXB plans (ΔMLD). No obvious trend was observed. We also studied the correlation between the ΔNTCP and the PTV size with all three LKB model parameter sets and with two different α/β ratios. No correlation was observed.

Although we could not find published literature to make direct comparisons against our current study on SBRT lung plans, it is relevant to mention previous studies on the influence of dose calculation algorithms on the predicted TCP and NTCP values [31, 43, 44], these studies revealed some potential differences in TCP/NTCP values depending on the calculation algorithm used. Nielsen et al. [43] showed an estimated NTCP value for pneumonitis that varied 4 % across the six investigated dose algorithms. Bufacchi et al. [44] reported that the NTCP value from AAA-calculated plans was lower than that from pencil beam-calculated plans in most treated sites. Petillion et al. [31] reported lower TCP and NTCP predictions when using advanced algorithms. Since our fractionation scheme and studied algorithms were much different from these published works, direct comparison cannot be meaningfully made between our findings and their results. The radiobiological indices impact of AAA and ABX dose computation algorithms were published by Rana et al. [45] and Padmanaban et al. [46]. The study of Rana et al concluded that both AAA and AXB predicted comparable NTCP and TCP values for low-risk prostate cancer plans. However, in Padmanaban et al. study on esophagus cancer, where it also involves complex tissue heterogeneities, a difference in TCP between 1.2 % and 3.1 % was found. The study of Petillion et al. [31] reported a 0.3 % lower TCP on breast in AXB plans compared with AAA plans.

It should be stated that there are large uncertainties in the biological models used and its associated parameters.The published TCP/NTCP model parameters that we used were obtained from studies that used different treatment techniques and dose algorithms from the present study. This would introduce some uncertainties too. In addition, some studies have suggested that the LQ model may overestimate the radiobiological effect at the dose level commonly used in SBRT [47]. Conversely, results from our group and others suggests that the LQ model may actually underestimate the cell killing expected at higher SBRT doses if a significant amount of vascular damage and indirect cell death occurs [48, 49]. Whatever the case, it certainly seems appropriate to only treat the findings of the current study as a relative comparison between the different dose calculation algorithms rather than studying the absolute expected values. There is likely a lot more biological information that could be added to the model to make it more truly a biological optimization and evaluation. As more clinical data are collected, it may help in the formulation of methods to predict biophysical response and result in more accurate predictions of TCP and NTCP.

## Conclusion

In this study, AXB-recalculated plans yielded lower TCP than the AAA-calculated plans. The lower TCP is associated with the lower PTV coverage in AXB-calculated plans. The maximum 11.9 Gy *EQD*_*2*_ dose of ΔD_95%_ in our patient cohort corresponds to up to 5.8 % ΔTCP for 30 months local control.AAA-calculated and AXB-recalculated plans yield very similar NTCP values. The above conclusion stays valid when different sets of published lung NTCP model parameters were used. No correlation was observed between the ΔTCP/ΔNTCP and the PTV size or location.

### Ethics approval and consent to participate

This study was approved by the University of Arkansas Medical Science Institutional Review Board (FWA00001119).

### Consent to publish

Not applicable.
